# Laminar Neural Field Model of Laterally Propagating Waves of Orientation Selectivity

**DOI:** 10.1371/journal.pcbi.1004545

**Published:** 2015-10-22

**Authors:** Paul C. Bressloff, Samuel R. Carroll

**Affiliations:** Department of Mathematics, University of Utah, Salt Lake City, Utah, United States of America; University of Tübingen and Max Planck Institute for Biologial Cybernetics, GERMANY

## Abstract

We construct a laminar neural-field model of primary visual cortex (V1) consisting of a superficial layer of neurons that encode the spatial location and orientation of a local visual stimulus coupled to a deep layer of neurons that only encode spatial location. The spatially-structured connections in the deep layer support the propagation of a traveling front, which then drives propagating orientation-dependent activity in the superficial layer. Using a combination of mathematical analysis and numerical simulations, we establish that the existence of a coherent orientation-selective wave relies on the presence of weak, long-range connections in the superficial layer that couple cells of similar orientation preference. Moreover, the wave persists in the presence of feedback from the superficial layer to the deep layer. Our results are consistent with recent experimental studies that indicate that deep and superficial layers work in tandem to determine the patterns of cortical activity observed *in vivo*.

## Author Summary

A major challenge in neurobiology is understanding the mechanisms underlying the formation and propagation of cortical waves and the underlying neural circuitry that supports them. A variety of neurological disorders such as epilepsy and spreading depression during migraine episodes are characterized by spatially localized oscillations and waves propagating across the surface of the brain. Moreover, traveling waves can be induced *in vitro* by electrically stimulating disinhibited cortical slices, and they are also observed *in vivo*. Physiological traveling waves are also correlated with perceptual waves such as those observed during binocular rivalry. Spatially structured neural network models (neural fields) are becoming increasingly popular as a method for exploring, both analytically and numerically, the conditions under which cortical traveling waves can occur. However, such models tend to ignore the laminar structure of cortex and the fact that sensory cortical cells are often tuned to particular features of a stimulus. In this study we develop a a laminar neural field model of propagating waves in primary visual cortex (V1), in which the orientation selectivity of V1 cells is taken into account. We demonstrate the important role of vertical inter-laminar connections and horizontal intra-laminar connections in supporting the propagation of orientation selective waves.

## Introduction

A major challenge in neurobiology is understanding the mechanisms underlying the formation and propagation of cortical waves and the underlying neural circuitry that supports them [1, 2]. A variety of neurological disorders such as epilepsy [3–5] and spreading depression [6–8] are characterized by spatially localized oscillations and waves propagating across the surface of the brain. Moreover, traveling waves can be induced *in vitro* by electrically stimulating disinhibited cortical slices [9–12] and are observed *in vivo*. Physiological traveling waves are also correlated with perceptual waves such as those observed during binocular rivalry [13–16]. Stimulus-induced and spontaneous laterally propagating waves *in vivo* have been observed in the primary visual cortex (V1) of anesthetized rodents [17–19], ferrets [20], cats [21–23], and primates [23, 24]. These observations have been obtained using various experimental methods, including optical imaging with voltage-sensitive dyes [17, 18, 22, 24], measurements of local field potentials (LFPs) [21, 23, 25], and calcium imaging [19, 20]. Two particular features of propagating activity in V1 motivate the modeling study of this paper. The first concerns the fact that most V1 cells respond preferentially to local stimuli with certain preferred properties such as orientation and left/right eye preference (ocular dominance). This means that propagation in cortical space is correlated with both retinotopy and stimulus feature preferences. Indeed, one can observe the lateral spread of orientation selectivity in carnivore V1 based on voltage-sensitive dye imaging [22], LFPs [21, 23] and epifluorescent imaging of calcium waves [20]. There is also indirect evidence for the propagation of orientation-dependent activity in V1 from experimental studies of binocular rivalry waves [13, 14]. The second feature concerns the laminar structure of V1, in particular, growing evidence that propagating activity in cortex is initially generated by local recurrent connections in deep (infragranular) layers, which then spreads vertically to superficial (supragranular) layers. This has been observed both in mouse V1 [19] and other cortical areas [26–28].

In this paper we develop a continuum neural field model of propagating waves in V1 that takes into account both the orientation-dependence of V1 neurons and the laminar structure of cortex. We focus on animals that have structured orientation preference maps such as ferrets, cats and primates rather than the “salt-and-pepper” organization found in rodents. That is, we take superficial layers of cortex to have a hypercolumnar structure consisting of orientation columns organized around a set of pinwheels [29–31], with strong local recurrent connections and weaker (modulatory) long-range horizontal connections that link neurons in different hypercolumns with similar orientation preferences [32–35]. Following several modeling studies [36–38], we assume that within each hypercolumn, neurons with sufficiently similar orientations tend to excite each other whereas those with sufficiently different orientations inhibit each other, and this serves to sharpen a particular neuron’s orientation preference. (Note, however, that the precise role of local recurrent connections in orientation tuning is still controversial, given the lack of direct evidence for antagonistic inhibition within cat V1 [39, 40].) Such a tuning mechanism suggests that local connections are structured with respect to orientation preference rather than retinotopy, and thus cannot provide a substrate for laterally propagating waves—this is also consistent with the observation of standing waves of orientation-dependent activity observed by Benucci et al. [21]. The weakness of the horizontal connections means that they also cannot support wave propagation on their own. In conclusion, the functional anatomy of superficial layers is consistent with experimental studies indicating that wave propagation is initiated in deep layers. Moreover, there is growing evidence that neurons in deep layers are more poorly tuned for orientation than those in superficial layers. For example layer 5 neurons in mouse V1 exhibit very little selectivity [41] and are weakly tuned in tree shrew [42]. Although orientation selectivity is observed in all layers within macaque V1, it appears to be weaker in deep layers [43, 44]. In the case of macaque, direct thalamic inputs from the lateral geniculate nucleus (LGN) are not orientation tuned, so that one possible source of weak orientation in deep layers is vertical feedback connections from input layer 4 or superficial layers. Note, however, that any orientation tuning in cat deep layers persists when feedback from superficial layers is suppressed [45]. If we assume that deep layer neurons are not strongly tuned for orientation, then it is possible that strong recurrent connections within the deep layers are spatially structured with respect to retinotopy rather than orientation preference, and thus support wave propagation.

The above experimental observations and hypotheses suggest that to a first approximation, propagating activity is initiated in orientation-independent deep layers, which subsequently induces the propagation of orientation-dependent waves in superficial layers via a combination of vertical inter-laminar connections and weak long-range horizontal connections within superficial layers. In this paper, we establish the validity of this emerging picture using a combination of mathematical analysis and numerical simulations. For simplicity, we consider a bilayer neural field model, which could loosely be interpreted in terms of an orientation-dependent superficial layer coupled to an orientation-independent deep layer. In our mathematical analysis, we construct traveling wave solutions in a simpler one-dimensional (1D) version of our model, and establish that inclusion of feedback from superficial to deep layer has little effect on wave propagation. We then show numerically that orientation selective traveling waves also occur in a two-dimensional (2D) model.

Finally, it should be noted that the laminar structure of V1 has been modeled extensively by Grossberg and collaborators [46–48]. These models are highly detailed and have been used to investigate a wide variety of phenomena in early visual processing. In contrast, our much simpler model is used to investigate large-scale cortical dynamics rather than visual information processing. There has been relatively little work on analyzing the dynamics of layered neural fields, one exception being recent work on the effects of inter-laminar coupling on regularizing wave propagation in stochastic neural fields [49–51], see also [52].

### Laminar Neural Field Model

One mathematical approach to studying cortical waves involves the analysis of continuum neural field models, in which the large—scale dynamics of spatially structured networks of neurons is described in terms of nonlinear integro-differential equations, whose associated integral kernels represent the spatial distribution of neuronal synaptic connections [53–58]. In this section we construct a neural field model of V1 that takes into account (i) the orientation selectivity of V1 neurons and (ii) the laminar structure of cortex.

**Fig 1:**
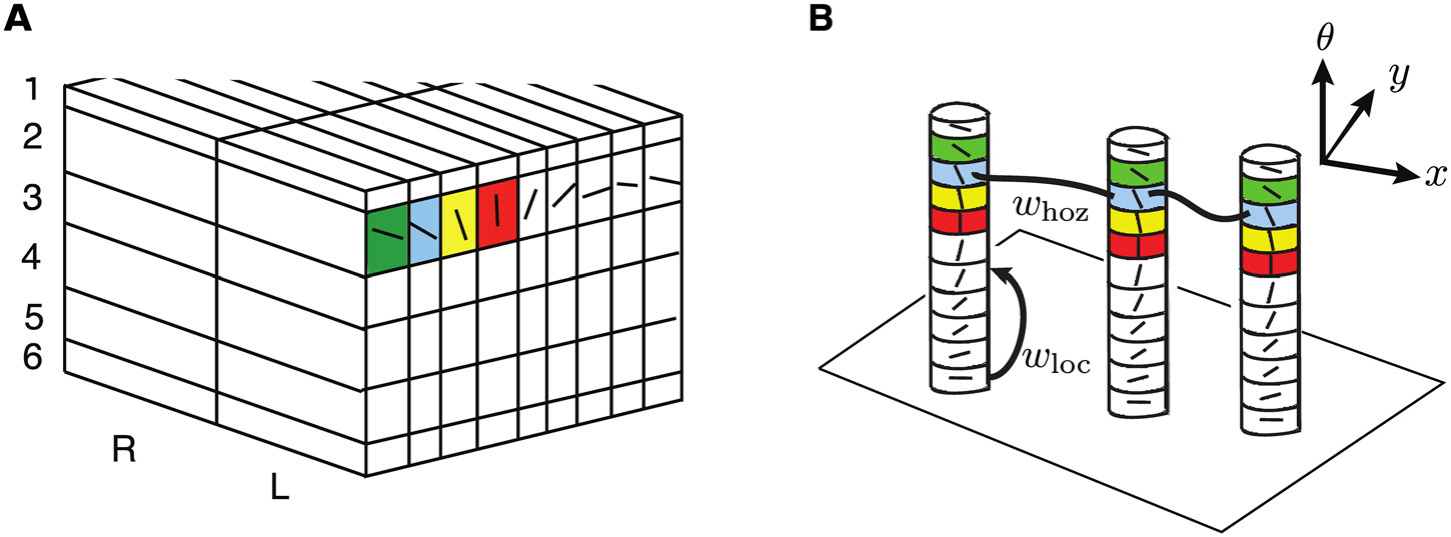
Schematic illustration of *R* ^2^ × *S* ^1^ model of V1. (a) Ice cube model of Hubel and Wiesel [59] showing cortical layers, orientation hypercolumns, and left(L)/right(R) ocular dominance columns. (b) Abstraction of superficial layers 2/3 as a collection of vertical cylinders (hypercolumns) that form a fibration of the plane. Patchy horizontal connections *w*_hoz_ are represented as links between the vertical cylinders. There are also local recurrent connections *w*_loc_ within each hypercolumn.

In order to motivate our model, we first briefly recall the original *ice cube* model of V1 due to Hubel and Wiesel [59], see Fig 1a. The latter treats superficial layers of V1 as a collection of horizontally arranged orientation hypercolumns. Each hypercolumn consists of cells with similar spatial (retinotopic) coordinates *x*, which can be further partitioned into columns consisting of cells with similar orientation preference *θ* (highlighted by different colors in Fig 1). This structure can be modeled by collapsing each hypercolumn into a single point (through some form of spatial coarse-graining) and treating V1 as a continuum of hypercolumns [38, 60, 61]. Thus neurons are lndexed by the pair {*x*, *θ*} with *x* ∈ ℝ^2^ labeling the hypercolumn at (coarse-grained) position *x* and *θ*, −*π*/2 < *θ* ≤ *π*/2, labeling the orientation preferences of neurons within the hypercolumn This so-called *R* ^2^ × *S* ^1^ structure of the superficial layers can be made more explicit by representing orientation hypercolumns as vertically drawn cylinders, which collectively form a fibration of the retinotopic plane (Fig 1b), see [62, 63]. (These abstract vertical columns should not be confused with the actual vertical columns passing through the various cortical layers.) If the synaptic weights within each orientation hyperolumn are taken to be *π*-periodic (topology of a circle), then one can treat each hypercolumn as a ring network that can amplify weakly tuned inputs from the thalamus or deeper cortical layers to form population based tuning curves [36, 37]. On the other hand, patchy horizontal connections between hypercolumns can be represented as links between similar orientation columns within different hypercolumns.

**Fig 2:**
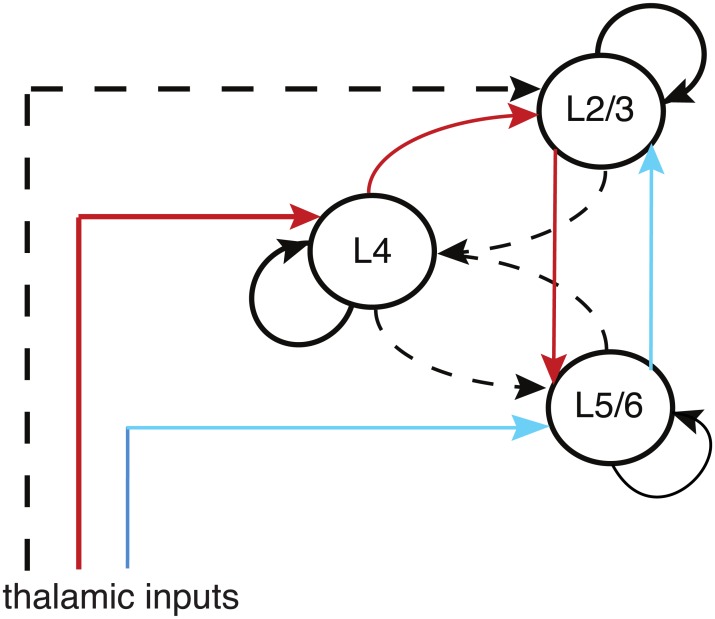
Schematic diagram illustrating an updated version of the classical microcircuit introduced by Douglas and Martin [68]. This minimal circuitry comprises superficial (layers 2 and 3) and deep (layers 5 and 6) pyramidal cells and a population of smooth cells in layer 4C. Feedforward thalamic inputs target all cell populations. Weaker (modulatory) connections are shown in gray. In the original microcircuit model it was assumed that direct thalamic inputs to deep layers were weak. However, a recent experimental study indicates that these inputs are comparable to those innervating layer 4C [67]. Thus the classical direct pathway *Th* → *L*4 → *L*2/3 → *L*5/6 (red) is supplemented by an additional direct pathway *Th* → *L*5/6 → *L*2/3 (blue).

Another feature of the original ice cube model is that the vertical distribution of stimulus features across different cortical layers is taken to be uniform so that to a first approximation the layered structure of cortex can be ignored. However, it has subsequently been shown that in non-human primates, for example, different cortical layers exhibit different properties [64–66]. Roughy speaking, the layers can be grouped into input layer 4, which is the main input layer from the thalamus, superficial layers 2, 3 that project to higher cortical regions, and deep layers 5, 6 that project back to thalamus and the brain stem. There is also extensive communication between the layers via so-called vertical connections. This is encapsulated by the modified canonical microcircuit shown in Fig 2. It has traditionally been assumed that the major feedforward pathway from thalamus is *LGN* → *L*4 → *L*2/3 → *L*5/6 with *L*5/6 sending feedback projections to *L*2/3. However recent experimental work on rat barrel cortex has established that there is a strong direct input from thalamus to *L*5/6 [67]. This suggests that there could be a second feedforward pathway *LGN* → *L*5 → *L*2/3 with respect to which *L*2/3 → *L*5 is viewed as feedback. Although the situation in the visual cortex of primates is less clear, we will explore the potential consequences of this second feedforward pathway for the propagation of orientation-selective waves.

**Fig 3:**
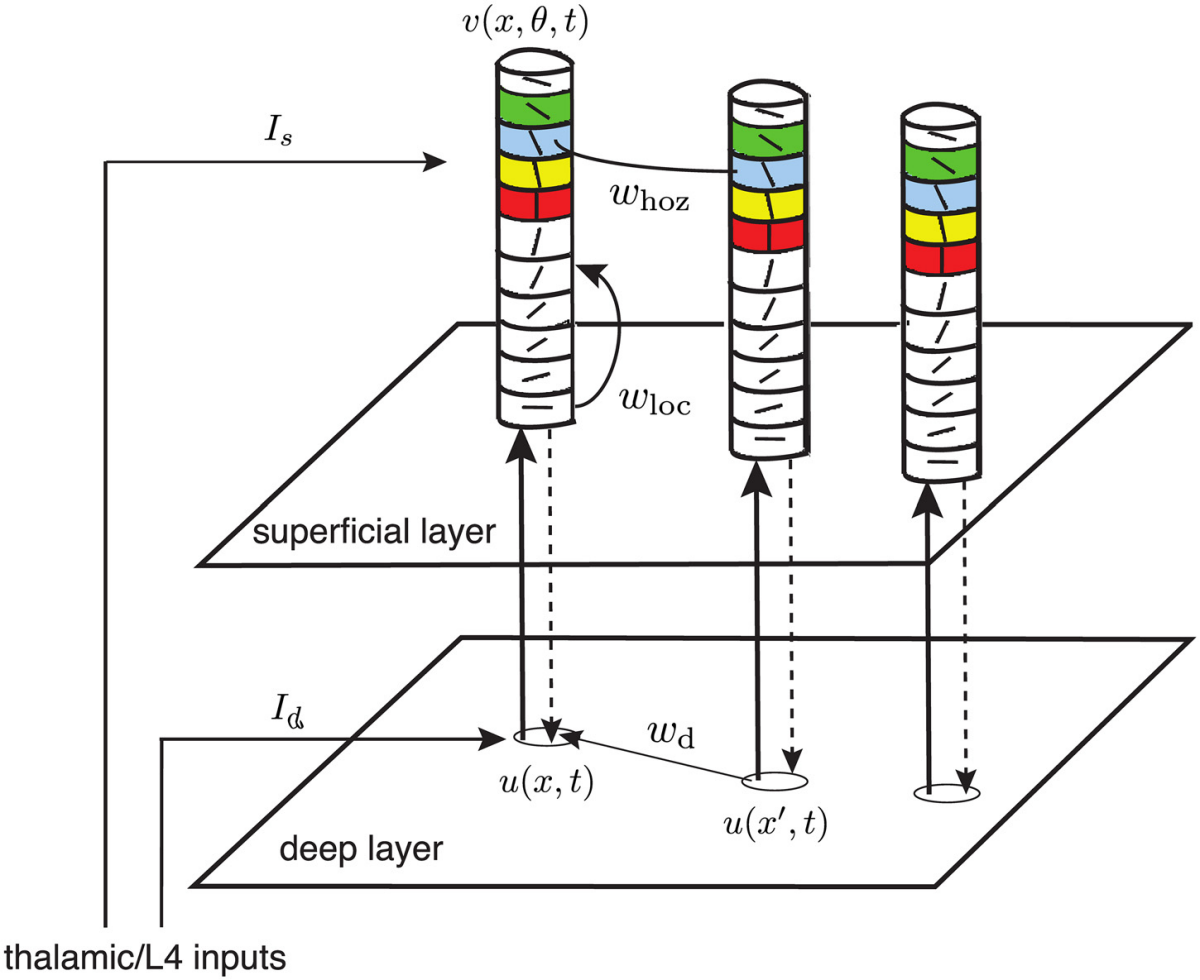
Schematic illustration of the laminar neural field model. Thalamic inputs feed into both the deep (*d*) and superficial (*s*) layers. At retinotopic position *x*, there is orientation independent activity *u*(*x*, *t*) in the deep layer and orientation-dependent activity *v*(*x*, *θ*, *t*) in the superficial layer. Intracortical connections in the deep layer are denoted by *w*_*d*_, whereas the intracortical connections *w* in the superficial can be decomposed into local *w*_loc_ and long-range parts *w*_hoz_. Vertical feedforward and feedback connections between the layers are also shown. Since we are interested in the self-sustained propagation of activity, we drop any external inputs *I*_*d*_ and *I*_*s*_ from thalamus or layer 4.

In this paper, we consider a bilaminar neural field model representing an orientation-dependent superficial layer coupled to an orientation-independent deep layer. Note that neurons in deep layers of V1 tend to be more weakly tuned for orientation than neurons in superficial layers [41, 42, 44]. This tuning could be even weaker at the population level if the distribution of orientation preferences is more disordered in deep layers. As a first approximation, we treat the deep layer as non-orientation selective. However, the main point is not that deep layer neurons are independent of orientation, but that recurrent connections within the deep layer are untuned to orientation. A schematic diagram of the basic network architecture is shown in Fig 3, which combines the ℝ^2^ × *S* ^2^ product structure of Fig 1 with the canonical microcircuit shown in Fig 2. The corresponding neural field equations take the form

$$\tau_{d}\partial_{t}u\left(x,t\right)=-u\left(x,t\right)+\int_{{\mathbb{R}}^{2}}w_{d}\left(x-x^{\prime }\right)f_{d}\left(u\left(x^{\prime },t\right)\right)dx^{\prime }+\gamma_{s}\int_{-\pi /2}^{\pi /2}f_{s}\left(v\left(x,\theta ,t\right)\right)d\theta$$ (1a)

$$\tau_{s}\partial_{t}v\left(x,\theta ,t\right)=-v\left(x,\theta ,t\right)+\int_{{\mathbb{R}}^{2}}\int_{-\pi /2}^{\pi /2}w(x,\theta |x^{\prime },\theta^{\prime })f_{s}\left(v\left(x^{\prime },\theta^{\prime },t\right)\right)d\theta^{\prime }dx^{\prime }+\gamma_{d}f_{d}\left(u\left(x,t\right)\right)$$ (1b)

Here *u*(*x*, *t*) denotes orientation-independent activity in the deep layer and *v*(*x*, *θ*, *t*) is the orientation-dependent activity in the superficial layer. The recurrent weight distributions in the two layers are denoted by *w*_*d*_ and *w*, respectively, and the corresponding relaxation time constants are given by *τ*_*d*_ and *τ*_*s*_. We fix the units of time by setting *τ*_*d*_ = 1 (typical physical values of *τ*_*d*_ are 1–10 ms). We also include vertical feedforward input from deep to superficial layers with strength *γ*_*d*_ and feedback from the superficial to the deep layer with strength *γ*_*s*_; in the latter case we average with respect to the orientation preference of neurons in the superficial layer. This is partly motivated by the observation that removing feedback does not appear to affect orientation tuning in cat layer 5 [45]. If this observation is combined with the assumption that recurrent connections in deep layers are not tuned for orientation, then the main source of orientation selectivity in deep layers is due to an external drive from an oriented stimulus. In the case of self-sustained propagating activity, such an input is absent so we can neglect any orientation dependence in the deep layer. Finally, the firing rate functions *f*_*j*_ are taken to be sigmoidal,

$$\begin{array}{c} f_{j}\left(u\right)=\frac{1}{1+e^{-\eta_{j}\left(u-\kappa_{j}\right)}},\;j=d,s \end{array}$$ (2)

where *η*_*j*_ is the gain and *κ*_*j*_, *κ*_*j*_ > 0, is the threshold of the *j*th layer. In the high gain limit, *η*_*j*_ → ∞, we have *f*_*j*_(*u*) → *H*(*u*−*κ*_*j*_), where *H* is the Heaviside function.

In this paper, we primarily analyze traveling wave dynamics with a 1D network which has isotropic horizontal connections. However, we briefly present results for the more relevant 2D network with anisotropic horizontal connections. We therefore first decompose the recurrent weights of the superficial layer into short-range connections that depend only on the neurons’ orientation preferences, and long-range patchy horizontal connections between compatible neurons:

$$\begin{array}{c} w(x,\theta |x\prime ,\theta \prime )=w_{loc}\left(\theta -\theta \prime \right)\delta \left(x-x\prime \right)+w_{s}\left(x-x\prime \right)w_{hoz}\left(\theta -\theta \prime \right), \end{array}$$ (3)

with *w*_loc_ and *w*_hoz_ even, *π*-periodic functions of *θ*, and *δ*(*x*) the Dirac delta function. The *θ*-dependent part of the horizontal connections, *w*_hoz_, is taken to be positive and narrowly peaked around zero so that only neurons with similar orientation preferences excite each other. Finally, *w*_*s*_(*x*−*x*′), determines the strength of connections between two populations at *x*, *x*′ and is assumed to depend on the spatial separation ∣*x*−*x*′∣. (For simplicity, we ignore any anisotropy in the horizontal connections.) Substituting the particular decomposition of *w* into Eqs (1a) and (1b) yields

$$\partial_{t}u\left(x,t\right)=-u\left(x,t\right)+w_{d}\left(x\right)\circ f_{d}\left(u\left(x,t\right)\right)+\gamma_{s}\int_{-\pi /2}^{\pi /2}f_{s}\left(v\left(x,\theta ,t\right)\right)d\theta$$ (4a)

$$\tau_{s}\partial_{t}v\left(x,\theta ,t\right)=-v\left(x,\theta ,t\right)+w_{loc}\left(\theta \right)*f_{s}\left(v\left(x,\theta ,t\right)\right)+w_{s}\left(x\right)\circ w_{hoz}\left(\theta \right)*f_{s}\left(v\left(x,\theta ,t\right)\right)+\gamma_{d}f_{d}\left(u\left(x,t\right)\right),$$ (4b)

where *x* ∈ ℝ and

$$\begin{array}{ccc} g\left(x\right)\circ h\left(x,t\right) & = & \int_{{\mathbb{R}}^{n}}g\left(x-x\prime \right)h\left(x\prime ,t\right)dx\prime ,\\ g\left(\theta \right)*h\left(x,\theta ,t\right) & = & \int_{-\pi /2}^{\pi /2}g\left(\theta -\theta \prime \right)h\left(x,\theta \prime ,t\right)d\theta \prime . \end{array}$$

One final aspect of our neural field model is that we do not explicitly model distinct excitatory and inhibitory populations. This is a common simplification of neural fields, in which the combined effects of excitation and inhibition are incorporated using, for example, Mexican hat functions [56, 58]. Typically, wave phenomena are modeled using excitatory neural fields, motivated by the fact that waves *in vitro* are observed in disinhibited cortical slices [9–12] and certain types of epilepsy appear to involve reductions in inhibition. On the other hand, the formation of population orientation tuning curves (stationary orientation bumps) in the ring model is modeled using a combination of short-range excitation and longer-range inhibition [36, 37]. Long-range horizontal connections are mediated by the axons of excitatory pyramidal neurons. However, they innervate both pyramidal neurons and feedforward interneurons so that they can effectively be treated as either excitatory or inhibitory, depending on stimulus conditions [35, 69, 70]. As a final level of complexity, there is a diversity of interneurons with different afferents and efferents distributed across different cortical layers [71]. In this paper we make the following simplifying assumptions with regards excitation and inhibition:

1. Intracortical connections in the deep layer are excitatory so that *w*_*d*_(*x*) is a positive function of *x*. (One could use a Mexican hat function, provided that the inhibition is not too strong to prevent wave propagation.)
2. Long-range horizontal connections in the superficial layer are excitatory so that *w*_*s*_(*x*) is a positive function of *x*.
3. The local connections *w*_loc_(*θ*) in the superficial layer are excitatory for small *θ* (similar orientations) and inhibitory for larger *θ* (Mexican hat function).
4. The vertical connections between the deep and superficial layers are excitatory so *γ*_*s*_, *γ*_*d*_ > 0.

## Results

One of the particularly useful aspect of continuum neural field models is that one can combine numerical simulations with analytical approaches adapted from the theory of partial differential equations [2]. In particular, the mathematical analysis allows us to identify parameter regimes where we expect traveling waves to occur, and to determine how the wave speed depends on these parameters. However, in order to carry out the mathematical analysis, one has to make some additional simplifying assumptions. Therefore, we proceed by initially considering a 1D version of our laminar model given by Eqs (4a) and (4b), in which feedback from the superficial to the deep layer is neglected (*γ*_*s*_ = 0). As a final simplification, we follow Amari [55] by taking the high gain limit, *η* → ∞, so that *f*_*j*_(*u*) → *H*_*j*_(*u*) ≡ *H*(*u*−*κ*_*j*_), where *H* is the Heaviside function. Following our mathematical construction of traveling wave solutions, we show numerically that the resulting waves persist in the presence of feedback and for high gain smooth firing rate functions. Technical aspects of the analysis are presented in **Materials and Methods**, including a proof that feedback has no affect on the existence and speed of the traveling wave in the Heaviside case. Having developed our theory for 1D waves, we then show numerically how analogous waves occur in the full 2D network model. Note that throughout this section we ignore any external inputs (*I*_*s*_ = *I*_*d*_ = 0 in Fig 3), since we are interested in the self-sustained propagation of activity due to recurrent connections rather than activity driven explicitly by a propagating external input. Thus, we assume that there are only transient inputs, which initiate the wave by determining appropriate initial conditions.

**Fig 4:**
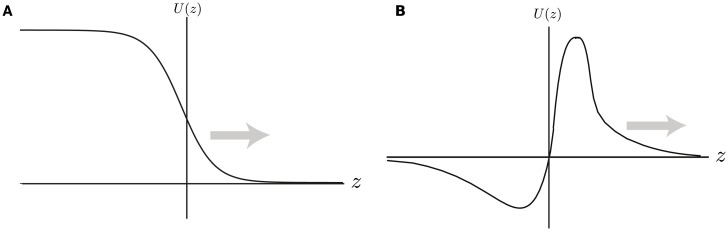
Schematic illustration of two types of traveling wave. (a) A traveling front. (b) A traveling pulse.

We proceed with our analysis with feedback connections removed, *γ*_*s*_ = 0. However, we show in **Materials and Methods** that our results are unchanged when including feedback. In the absence of feedback, the deep layer is independent of activity in the superficial layer and Eq (4a) reduces to the classical scalar neural field equation

$$\begin{array}{c} \partial_{t}u\left(x,t\right)=-u\left(x,t\right)+w_{d}\left(x\right)\circ H_{d}\left(u\left(x,t\right)\right). \end{array}$$ (5)

The standard analysis of Amari [55] can then be used to establish the existence of a traveling wave solution in the deep layer. Formally speaking, a traveling wave is a solution that travels at constant velocity and fixed shape. For one-dimensional systems, one can distinguish two types of solitary traveling wave: a traveling front linking a stable *down state* to a stable *up state*, and a traveling pulse that begins and ends at the down state, see Fig 4. In this paper, we focus on traveling fronts. (In order to obtain traveling pulses, it would be necessary to include some form of local negative feedback such as spike frequency adaptation or synaptic depression [72].) Suppose that the excitatory weight distribution *w*_*d*_(*x*) is an even function of *x* and is monotonically decreasing for *x* ≥ 0. A common choice is the exponential weight distribution

$$\begin{array}{c} w_{d}\left(x\right)=\frac{{\bar{w}}_{d}}{2\sigma_{d}}e^{-\left|x\right|/\sigma_{d}}, \end{array}$$ (6)

where *σ*_*d*_ determines the range of synaptic connections. The latter tends to range from 100 *μ*m to 1 mm. Mathematically speaking, a traveling front solution of Eq (5) takes the form *u*(*x*, *t*) = *U*(*z*) for some fixed profile function *U*, where *z* = *x* − *ct* is a traveling wave coordinate and *c* denotes the wavespeed. We also require that

$$\lim_{z\to -\infty }U\left(z\right)=w_{d,0}>\kappa_{d},\;\lim_{z\to \infty }U\left(z\right)=0$$

such that *U*(*z*) only crosses the threshold *κ*_*d*_ once. Since Eq (5) is translation invariant in space, we are free to take the threshold crossing point to be at the origin, *U*(0) = *κ*_*d*_, so that *U*(*z*) < *κ*_*d*_ for *z* > 0 and *U*(*z*) > *κ*_*d*_ for *z* < 0. Substituting the traveling front solution into Eq (5) and requiring the resulting solution to be bounded then yields an implicit formula for the wave speed given by

$$\begin{array}{c} \kappa =\frac{1}{\left|c\right|}\int_{0}^{\infty }e^{-y/|c|}W_{d}\left(\text{sign}\left(c\right)y\right)dy,\;W_{d}\left(y\right)=\int_{y}^{\infty }w_{d}\left(x\right)dx. \end{array}$$ (7)

One of the useful aspects of the above analysis that it allows us to derive an explicit expression for the wavespeed as a function of physiological parameters such as firing threshold and range of synaptic connections. In the case of the exponential weight distribution (6), the relationship between wavespeed *c* and threshold *κ*_*d*_ is

$$c=\frac{\sigma_{d}}{2}\left[{\bar{w}}_{d}/\kappa_{d}-2\right]>0,\text{ for }\kappa_{d}<\frac{{\bar{w}}_{d}}{2},$$ (8a)

$$c=\frac{\sigma_{d}}{2}\frac{{\bar{w}}_{d}/\kappa_{d}-2}{{\bar{w}}_{d}/\kappa_{d}-1}<0,\text{ for }\frac{{\bar{w}}_{d}}{2}<\kappa_{d}<{\bar{w}}_{d}.$$ (8b)

This establishes the existence of a unique front solution for fixed *κ*_*d*_, which travels to the right (*c* > 0) when $\kappa_{d}<{\bar{w}}_{d}/2$ and travels to the left (*c* < 0) when ${\bar{w}}_{d}>\kappa_{d}>{\bar{w}}_{d}/2$. If the threshold *κ*_*d*_ is too large or the recurrent connections are too weak so that ${\bar{w}}_{d}<\kappa_{d}$, then no traveling wave exists. It can be proven that the traveling front is stable [73]. Moreover, given the existence of a traveling front solution for a Heaviside rate function, it is possible to extend the analysis to a smooth sigmoid nonlinearity using a continuation method [74]. Finally, reintroducing the time constant *τ*_*d*_, we see from Eq (8b) that the wavespeed has the order of magnitude *c* ∼ *σ*_*d*_/*τ*_*d*_ = 10 cm/s for *σ*_*d*_ = 1 mm and *τ*_*d*_ = 10 msec. In the following we fix the units of space and time by setting *σ*_*d*_ = 1 = *τ*_*d*_.

### No spatially coherent orientation selective waves in the absence of horizontal connections (*w*_*s*_ = 0)

Now suppose that the traveling front solution of the deep layer drives activity in the superficial layer via the vertical feedforward connections. For the moment, we ignore the effects of horizontal connections by setting *w*_*s*_ = 0. (From our analysis of waves in the deep layer, we know that weak horizontal connections cannot on their own sustain propagating activity in the superficial layer.) Eq (4b) then reduces to

$$\begin{array}{c} \tau \partial_{t}v\left(x,\theta ,t\right)=-v\left(x,\theta ,t\right)+w_{\text{loc}}\left(\theta \right)*H_{s}\left(v\left(x,\theta ,t\right)\right)+\gamma_{d}H\left(ct-x\right) \end{array}$$ (9)

where we have used *H*_*d*_(*u*(*x*, *t*)) = *H*(*U*(*x* − *ct*) − *κ*_*d*_) = *H*(*ct* − *x*) with *c* > 0. It is important to note that although Eq (9) and its generalization for *w*_*s*_ > 0 suggests one could eliminate the deep layer and simply drive the superficial layer explicitly with a propagating external input, two important features are lost. First, one needs to understand the intrinsic cortical mechanisms that support wave propagation (in this case recurrent excitation in the deep layer) and determine the corresponding wavespeed and second, we will later include the effects of feedback from the superficial to the deep layer. (In **Materials and Methods**, we show that the inclusion of feedback does not affect the wavespeed *c* in the deep layer so that Eq (9) still holds.) The neural populations labeled by spatial position *x* act independently of one another so that we effectively have a continuum of independent orientation tuning equations (ring models) with time-dependent input *γ*_*d*_ *H*(*ct*−*x*) for fixed *x*. Again, we can analyze each ring model using the standard analysis of Amari [55]. In spatial regions sufficiently far from the front at *x* = *ct*, we can assume that each ring network has reached a quasi-stationary state *V*(*θ*), which satisfies an equation of the form

$$\begin{array}{c} V\left(\theta \right)=w_{\text{loc}}\left(\theta \right)*H_{s}\left(V\left(\theta \right)\right)+\gamma_{d}\chi , \end{array}$$ (10)

with *χ* = 1 if *x* ≪ *ct* and *χ* = 0 if *x* ≫ *ct*. As shown by Amari [55], for certain choices of local recurrent connections, there exist stationary bump solutions, which in the present context can be interpreted as population-level orientation tuning curves. Conditions for the existence and stability of bumps can be determined in terms of the function

$$W\left(\theta \right)=\int_{0}^{\theta }w_{loc}\left(\theta \prime \right)d\theta \prime .$$

**Fig 5:**
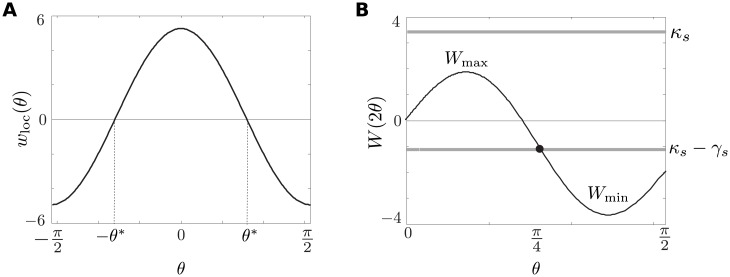
Existence of orientation bumps in the ring model. (a) Plot of Mexican hat local connections, *w*_loc_ = (-2 + 8 cos(2*θ*))/π. (b) Plot of *W*(2*θ*). The intercepts of *W*(2*θ*) with the horizontal line *y* = *κ*_*s*_ − *γ* determine the bump solutions in the presence of a constant input *γ*.

In particular, the existence condition for a symmetric bump centered about *θ* = 0 is

$$V\left(\theta \right)=W\left(\theta +\Delta \right)+W\left(\theta -\Delta \right)+\gamma_{d}\chi$$

where Δ is the half bump width satisfying *W*(2Δ) = *κ*_*s*_ − *γ*_*d*_, and the stability condition is *W*′(2Δ) < 0. The existence and stability of bumps can then be analyzed graphically as illustrated in Fig 5 for *w*_loc_ given by a cosine function:

$$\begin{array}{c} w_{\text{loc}}\left(\theta \right)=\left(w_{0}+w_{1}\cos\left(2\theta \right)\right)/\pi ,\;w_{0}+w_{1}>0>w_{0}-w_{1}. \end{array}$$ (11)

The latter realizes a basic assumption of the ring model [36, 37], namely, that neurons with similar orientation preferences excite each other, whereas those with sufficiently different orientation preferences inhibit each other.

In our model we assume that the superficial layer operates under the following conditions:

1. *W*_max_ < *κ*_s_—condition for the down state *V*(*θ*) ≡ 0 to be the only stable steady state when *χ* = 0.
2. There exists a unique stable bump solution *V*_bump_(θ) of half-width Δ_0_ when *χ* = 1. This holds provided that *W*_min_ < *k*_s_ − *γ*_d_ < *W*_max_.
3. *κ*_*s*_ − *γ*_*d*_ < 0—condition for the down-state not coexisting with the bump state when *χ* = 1.

Under these operating conditions, we then have the following scenario with regards to the response of the superficial layer to the propagating drive from the deep layer. Ahead of the front (*x* > *ct*) the superficial layer is in the down state. However, as soon as the front passes a particular location *x*, the down state disappears and the ring network at *x* evolves to a stationary bump solution at a relaxation rate determined by the time constant *τ*_*s*_. One subtle feature is that there has to be some orientation-dependent perturbations in the superficial layer, otherwise the activated network evolves to an orientation-independent state that fluctuates around the threshold *κ*_*s*_[75]. Such perturbations occur in the presence of horizontal connections, see below. However, since there are no horizontal connections linking distinct hypercolumns, each activated ring network forms a bump whose peak is uncorrelated with other bumps. Hence as activity propagates along the deep layer, connections to the superficial layer drive the activity to form orientation tuning curves with a random tuned orientation at each location *x*.

### Spatially coherent orientation selective waves in the presence of weak horizontal connections (*w*_*s*_ > 0)

**Fig 6:**
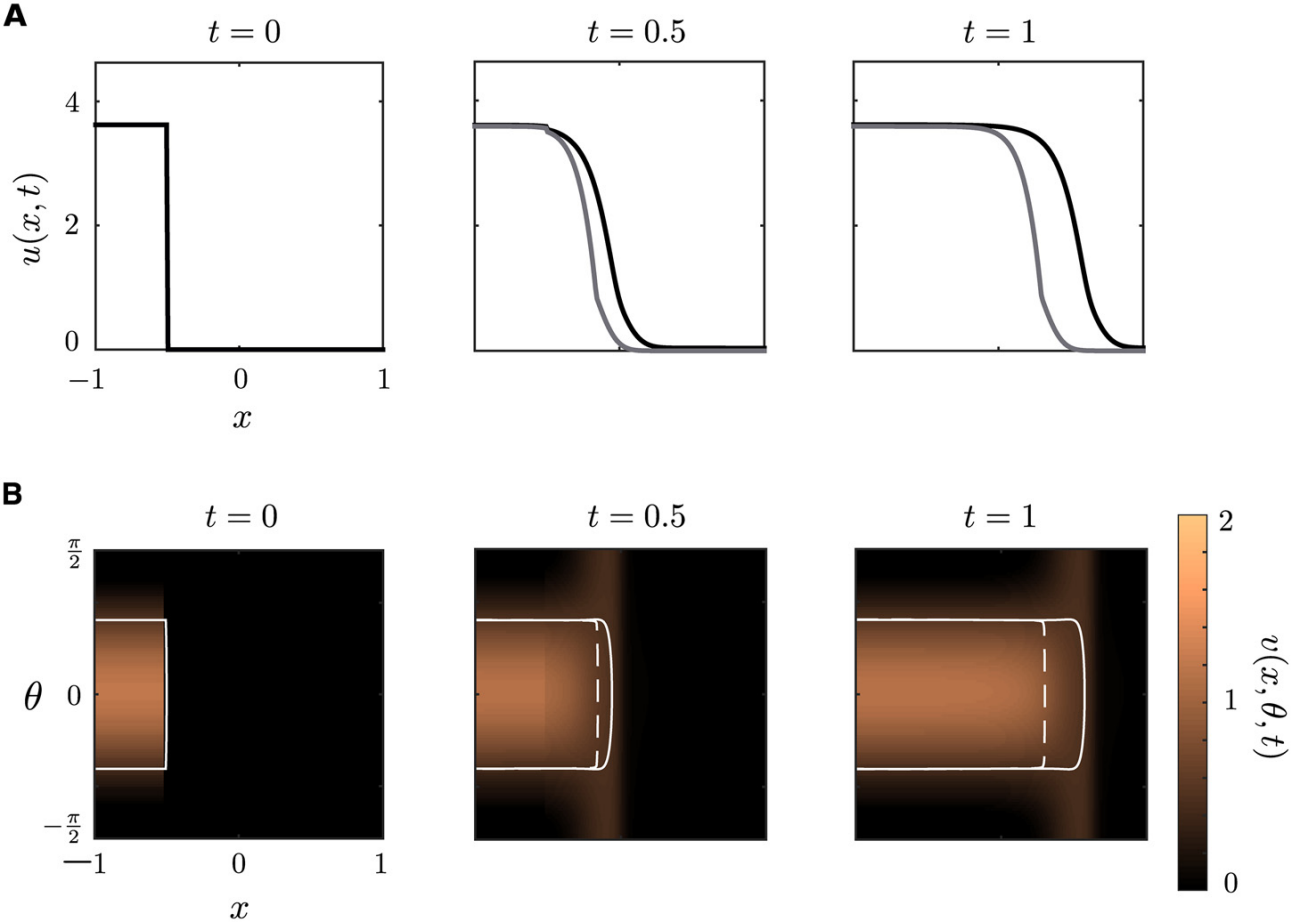
Numerical simulation of orientation-dependent traveling wave solution in the superficial layer. **(A)** Plot of deep layer traveling wave solution for both Heaviside firing rate (grey) and smooth firing rate (black). **(B)** Orientation-dependent traveling wave solution in superficial layer for smooth firing rate function. We superimpose the threshold crossing contour for both Heaviside firing rate (white dashed) and smooth firing rate (white solid). In each time frame we show a contour plot of activity *v*(*x*, *θ*, *t*) in the (*x*, *θ*)-plane with high (superthreshold) activity indicated by orange (light) and low (subthreshold) activity indicated by brown (dark). Parameter values: *w*_d,0_ = 2, *w*_s,0_ = 0.1, *w*_0_ = -1, *w*_2_ = 1, *k*_d_ = *k*_s_ = 0.5, *γ*_d_ = *γ*_s_ = 1, *σ*_d_ = 0.1, *σ*_s_ = 1, *τ*_d_ = *τ*_s_ = 0.1, *η* = 10.

**Fig 7:**
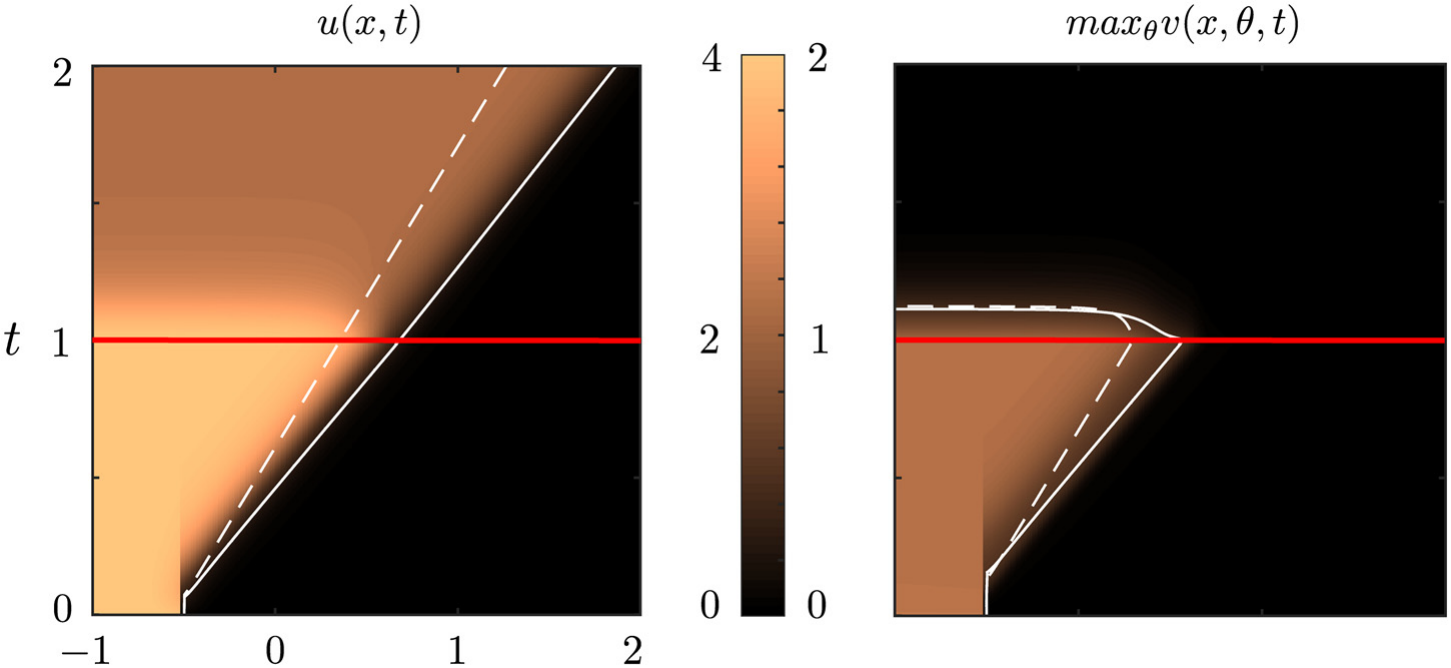
Propagation failure in the superficial layer in the absence of feedfoward connections. Density plot of traveling wave solutions in the (*x*, *t*) plane for deep and superficial layers before and after feedforward connections are removed at time *t* = 1 (indicated by the red line). For *t* > 1 the solution in the superficial layer decays to the rest state while the solution in the deep layer persists with the wave speed approximately unchanged indicating that the feedback connections play a negligible role in wave propogation. For comparison we superimpose a threshold crossing contour for both solutions with the Heaviside (dashed white line) and the smooth (solid white line) firing rate functions. Parameter values are the same as Fig 6.

We now establish that weak long-range horizontal connections serve to correlate the phase of the bump along the spatial direction leading to the propagation of a coherent orientation tuning curve. In Fig 6 we show numerical simulations of the full system given by Eqs (4a) and (4b), which includes feedback from the superficial layer to the deep layer, with the initial conditions

$$u\left(x,\theta ,0\right)=\left(w_{d,0}+2\gamma_{s}\Delta \right)H\left(x_{0}-x\right),\;v\left(x,\theta ,0\right)=V_{\text{bump}}\left(\theta \right)H\left(x_{0}-x\right).$$

where *V*_bump_(*θ*) is the stationary bump solution of half-width Δ. We take the various weight distributions to have the particular form

$$\begin{array}{ccc} w_{d/s}\left(x\right) & = & \frac{w_{d/s,0}}{\sqrt{2\pi \sigma_{d/s}^{2}}}e^{-x^{2}/2\sigma_{d/s}^{2}}\\ w_{\text{loc}} & = & \left(w_{0}+2w_{2}\cos\left(2\theta \right)\right)/\pi ,\;w_{\text{hoz}}=\left(1+\cos\left(2\theta \right)\right)/\pi \end{array}$$

with parameter values specified in the caption of Fig 6. Note, however, that our results are insensitive to the precise choice of weight functions, provided they satisfy the various conditions highlighted in the text. In Fig 6, we plot the deep layer activity *u*(*x*, *t*) along side a density plot of the superficial layer activity *v*(*x*, *θ*, *t*) in the (*x*, *θ*) plane at successive times *t* for both a Heaviside and smooth firing rate function. Each snapshot clearly shows a sharp front separating an orientation bump centered about *θ* = 0 to the left of the front and an orientation-independent low activity state (down state) to the right of the front. Moreover, the front itself propagates to the right in successive frames. Additionally, we see that the results are qualitatively the same in the smooth firing rate case, except that the wave speed is larger. Now suppose that we simulate Eqs (4a) and (4b) as in Fig 6, and allow the solution to evolve for one time unit, *t* = 1. At this point, we remove feedforward connections from the deep to the superficial layer and allow the solution to evolve for another time unit. In Fig 7 we show a density plot of both the deep and superficial layer solutions in the (*x*, *t*) plane (we take max_*θ*_ *v*(*x*, *θ*, *t*)). We see that after the feedfoward connections are removed, the activity in the superficial layer decays towards the rest state, while the activity in the deep layer persists as a traveling wave. This implies that input from the deep layer to the superficial layer is necessary for wave propagation, which is consistent with the various experimental studies highlighted in the **Introduction** [19, 26–28]. Furthermore, we see that the wave speed for the deep layer is approximately unchanged indicating that feedback connections to the deep layer are both unnecessary and negligible for wave propagation in the deep layer, which agrees with our analysis for Heaviside firing rates in **Materials and Methods**.

**Fig 8:**
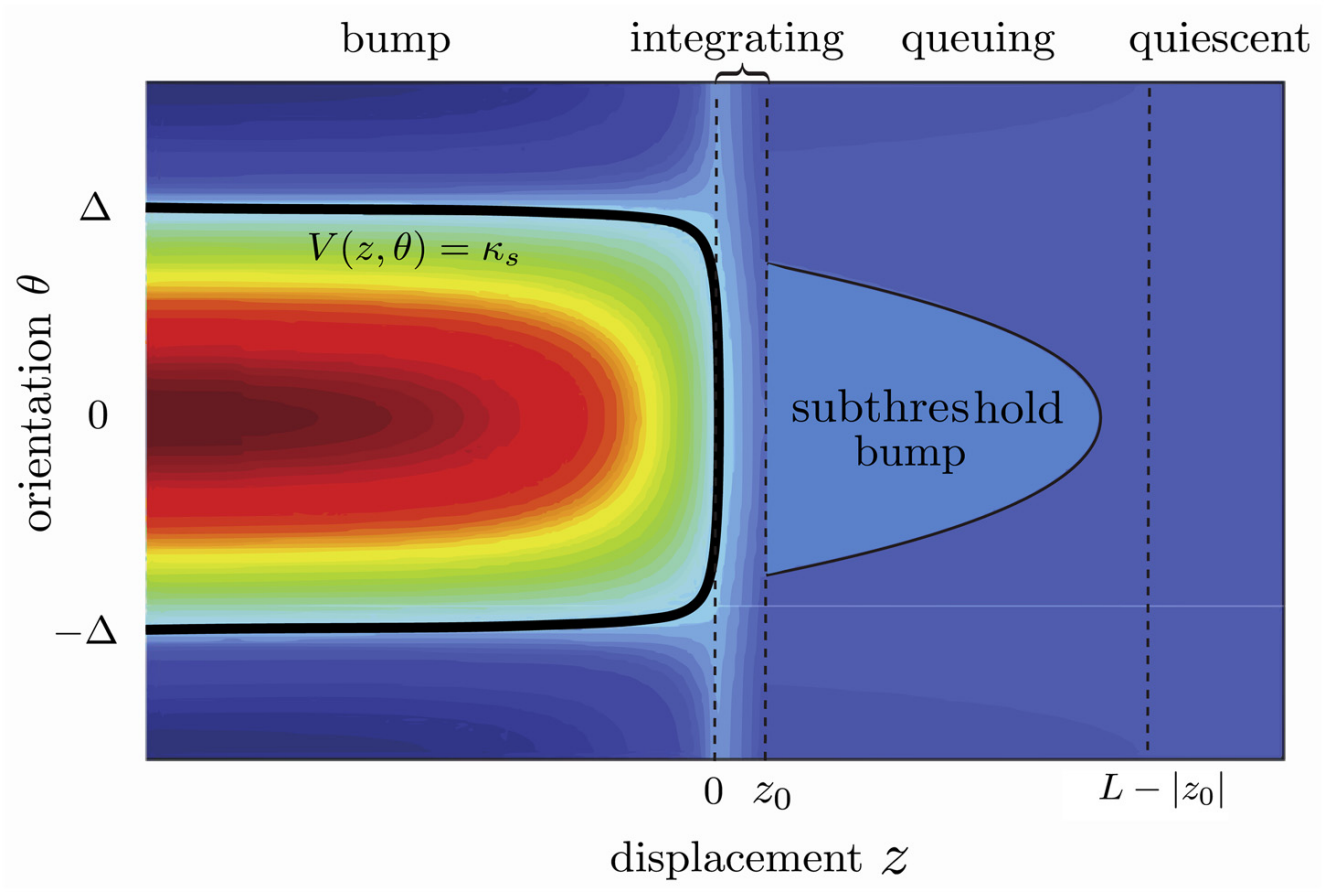
Graphical construction of a traveling wave solution. The four regions *quiescent, queuing, integrating, bump* are superimposed on a traveling wave solution of the neural field Eq (12). Here *L* denotes the range of long-range connections and *z*_0_, *z*_0_ < 0 is the point at which activity in the superficial layer first reaches threshold. (The location of the front in the deep layer is at *z* = 0.) In the quiescent region (*z* > *L* − ∣*z*_0_∣) there is no activity. In the queuing region (0 < *z* < *L* − ∣*z*_0_∣) neural populations have not received input from the deep layer, but receive a weak orientation-dependent input from the active region via the horizontal connections. This leads to the formation of sub-threshold orientation bumps, which grow in the integrating region (*z*_0_ < *z* < 0) where populations now also receive orientation-independent input from the deep layer. Finally, the growing bumps cross threshold in the bump region (*z* < *z*_0_) evolving to a bump of half-width Δ as *z* → −∞. (We use a different color code than Figs 6 and 7 in order to highlight the different regions. Here red indicates high activity and blue low activity.)

Inclusion of horizontal connections yields the following generalization of Eq (9):

$$\tau_{s}\partial_{t}v\left(x,\theta ,t\right)=-v\left(x,\theta ,t\right)+w_{loc}\left(\theta \right)*H_{s}\left(v\left(x,\theta ,t\right)\right)+w_{s}\left(x\right)\circ w_{hoz}\left(\theta \right)*H_{s}\left(v\left(x,\theta ,t\right)\right)+\gamma_{d}H\left(ct-x\right)$$ (12)

with *c* independent of the feedback and determined solely by the deep layer. Unfortunately, it is not possible to obtain an explicit traveling wave solution of Eq (12). However, we observe that there are four distinct regions of interest: *quiescent, queuing, integrating, bump*. This is illustrated in Fig 8, where we superimpose these regions on a density plot of activity in the moving frame for the traveling wave solution. That is, we set *v*(*x*, *θ*, *t*) = *V*(*z*, *θ*) with *z* = *x* − *ct* and plot *V*(*z*, *θ*) in the (*z*, *θ*)-plane. This graphical construction can be used to qualitatively describe the traveling wave solution, as detailed in **Materials and Methods**. In order to define the different regions we introduce two quantities: the range *L* of long-range connections and the point *z*_0_ at which activity in the superficial layer first reaches the threshold *κ*_*s*_, given that the front in the deep layer is located at *z* = 0. We find that if *w*_s,0_ is sufficiently small, so that the superficial layer does not support wave propagation on its own, then −*L* < *z*_0_ < 0 necessarily. The quiescent region is where neurons receive no synaptic inputs from either the deep layer or from active areas of the superficial layer, since they are out of range of the corresponding horizontal connections. Since the zero state is the only stable state when there is no input from the deep layer we find that *V*(*z*, *θ*) = 0 for *z* > *L*−∣*z*_0_∣ > 0. In the queuing region *z*_0_ < *z* < *L*−∣*z*_0_∣ neural populations have not yet received input from the deep layer, but have received a weak orientation dependent input from the active region via the horizontal connections. This leads to the formation of sub-threshold orientation bumps, which cannot cross threshold since the horizontal connections are weak. On the other hand, in the integrating region *z*_0_ < *z* < 0, neurons also receive input from the deep layer. Following our discussion on the existence of bumps, the input from the deep layer places the network in a parameter regime where the only stable solution is a bump. Hence the populations start integrating towards threshold as sub threshold bumps. Finally for *z* < *z*_0_, the populations continue to integrate towards the steady state bump solution *V*_bump_(*θ*).

### Traveling waves in 2D

As a starting point to understanding the dynamics of traveling waves of orientation selectivity in 2D, we numerically solve the full neural field Eqs (1a) and (1b). Analogous with the results in 1D we find that feedback connections do not affect the wave speed or the threshold crossings. Therefore, suppose that *u*(*x*, *t*) is a solution to Eq (1a) such that *u*(*r*, *t*) > *κ*_*d*_ for *r* = (x, y) ∈ *U*(*t*) ⊂ ℝ^2^ and *u*(*r*, *t*) < *κ*_*d*_ for *r* ∉ *U*(*t*), where *U*(*t*) is some set which changes in time. Hence

$$H_{d}\left(u\left(r,t\right)\right)=I_{U(t)}\left(r\right)$$

where $I_{U}$ is the indicator function for the set *U*. The dynamics for the the superficial layer then evolve according to

$$\begin{array}{c} \partial_{t}v\left(r,\theta ,t\right)=-v\left(r,\theta ,t\right)+\gamma I_{U\left(t\right)}\left(r\right)+\int_{{\mathbb{R}}^{2}}\int_{0}^{\pi }w(r,\theta |r\prime ,\theta \prime )H_{s}\left(v\left(r\prime ,\theta \prime ,t\right)\right)d\theta \prime dr\prime . \end{array}$$ (13)

Here we decompose the synaptic weight kernel as

$$\begin{array}{l} \begin{array}{ccc} w(r,\theta |r^{\prime },\theta^{\prime }) & = & w_{loc}\left(\theta -\theta \prime \right)\delta \left(r-r\prime \right)+w_{A}\left(r\prime -r,\theta \right)w_{hoz}\left(\theta -\theta \prime \right) \end{array}\\ \begin{array}{ccc} \;w_{A}\left(r,\theta \right) & = & \;w_{s}\left(\left|r\right|\right)A\left(\text{arg}\left(r\right)-\theta \right). \end{array} \end{array}$$

This is similar to the 1D case, except that we have included the function *A*(arg(*r*′−*r*)−*θ*) to account for anisotropies in the horizontal connections. Here arg(*r*) denotes the angle from the *x*-axis to the vector *r* and *A* is some positive, symmetric function, narrowly peaked about *θ* = 0, describing the strength of connections between neurons at locations *r* and *r*′ with common preferred orientation *θ*. That is, populations with similar orientation preferences in different hyper columns are only connected if they lie along the line with angle similar to their common orientation preference.

We would like to understand how anisotropic horizontal connections correlate the phase of the bump such that spatially structured patterns of orientation tuning emerge in the region *U*(*t*). Recently, we have studied this problem when the region *U* is fixed in time [75]. In this case we find that the solution inside the region *U* is approximately given by

v(r,θ,t)=V(θ-ϕ(r,t))

where *V*(*θ*) is the bump solution and *ϕ*(*r*, *t*) is the phase of the bump. We then derived an integro-differential equation describing the spatiotemporal dynamics of the phase of the bumps *ϕ*(*r*, *τ*) on the slow time scale *τ* = *ɛt*. We found that when the horizontal connections are taken to be isotropic, *A* ≡ 1, that the synchronous state *ϕ*(*r*) = *ϕ*_0_ is a steady state solution for any region *U*. However this is no longer necessarily the case when the connections are anisotropic, rather the geometry of the region and the structure of the anisotropy determine the solutions.

Our first example is the 2D analog of the 1D traveling wave solution namely, a plane wave. Note that the 2D neural field equation for the deep layer is

$$\partial_{t}u=-u\left(r,t\right)+w_{d}\left(r\right)\circ H_{d}\left(u\left(r,t\right)\right)$$

and thus if

$$\int_{-\infty }^{\infty }w_{d}\left(|r|\right)dy=w_{e}\left(|x|\right)$$

(an example of such a function is a Gaussian) we can find a solution *u* = *u*_*e*_(*x*, *t*) which is constant in the *y*-direction, given by

$$\partial_{t}u_{e}\left(x,t\right)=-u_{e}\left(x,t\right)+\int_{\mathbb{R}}w_{e}(|x-x\prime |)H_{d}\left(u_{e}\left(x\prime ,t\right)\right)dx\prime ,$$

which supports the 1D traveling front solution constructed above. By rotational symmetry of the system, it then follows that *u*(*r*, *t*) = *u*_*e*_(*R*_*ϕ*_ *r*, *t*) for any rotation by *ϕ*, *R*_*ϕ*_, is also a solution. This corresponds to a front which is constant along the vector **k** = (cos*ϕ*, −sin*ϕ*) and traveling in the direction **k** ^⊥^. One can then perform a similar simplification for the activity in the superficial layer. The simplest case is when the connections are isotropic, *A* ≡ 1, so that *v*(*r*, *θ*, *t*) = *v*_*e*_(*x*, *θ*, *t*) where *v*_*e*_ is a solution to Eq (9b). In this case any phase in the bump yields a valid solution. However this is no longer the case with anisotropic connections due to the fact that integrating out one direction will not get rid of the dependence of *w*_*A*_ on the orientation *θ*. Deriving an effective 1D equation for anisotropic connections is a non-trivial task so we leave this for future work. For now we numerically simulate the network with the full system in Eqs (4a) and (4b) with initial conditions

$$u\left(x,y,0\right)=H\left(x_{0}-x\right),\;v\left(x,y,\theta ,0\right)=0.$$

We choose to set *v* = 0 in order to see what the phase of the bumps naturally settle to.

**Fig 9:**
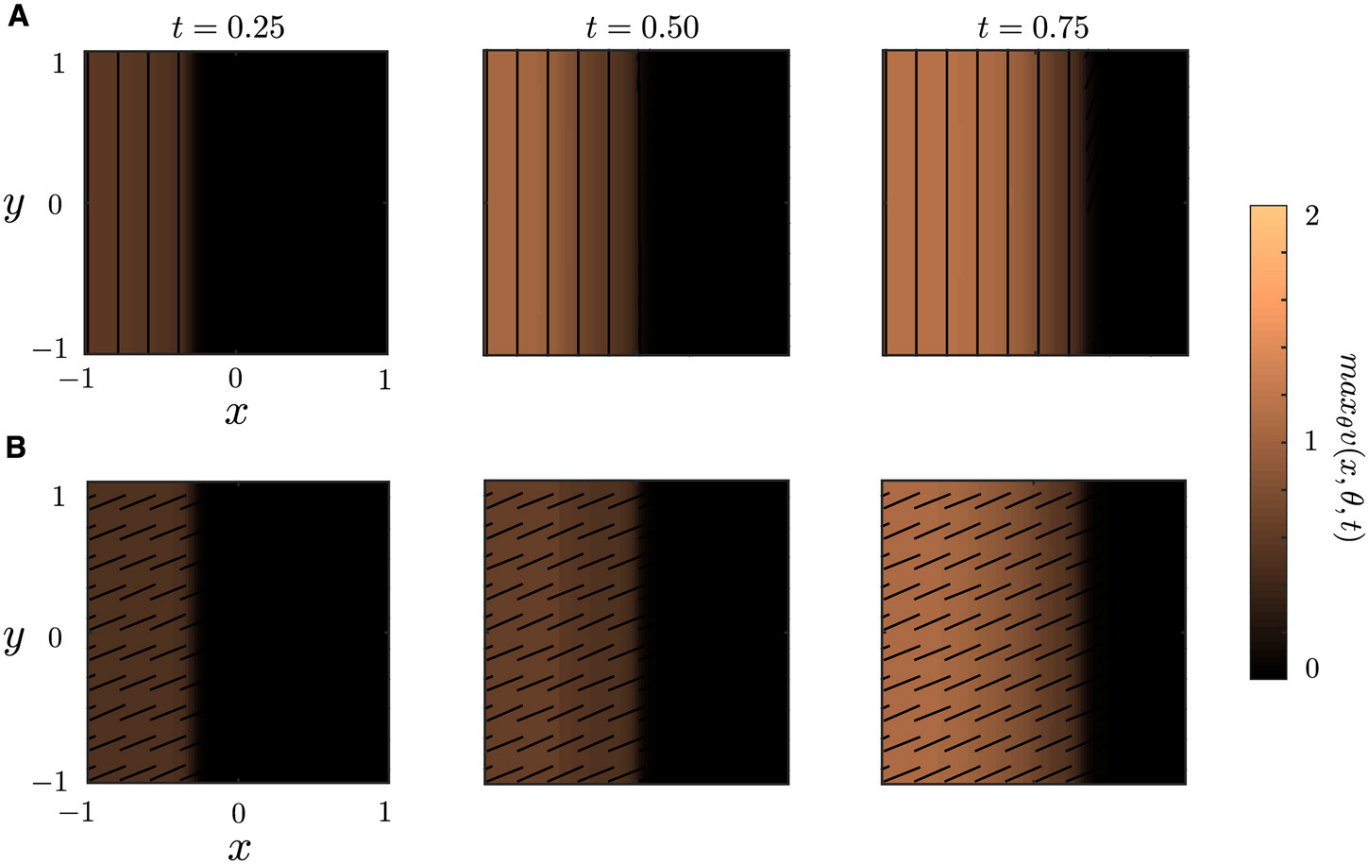
Two-dimensional plane waves. Numerical simulation of Eqs (4a) and (4b) for **(A)** anisotropic connection *A*(*θ*) = 1 + cos(2*θ*) and **(B)** isotropic connections *A* ≡ 1. All parameter values are the same as in Fig 6 and we take *x*_0_ = −0.5. All weight functions are the 2D analogs of the functions used for 1D.

In Fig 9 we see that with anisotropic connections the phase is parallel to the boundary of the front. In other words, the orientation is orthogonal to the direction of wave propagation. However with isotropic connections the phase tends towards some synchronous solution entirely unrelated to the boundary or direction of propagation, which is consistent with our analysis of 1D waves. Simulating the network repeatedly with noise we find that this synchronous phase is random. Although isotropic horizontal connections cause the network to have a synchronous phase, without anisotropy there is no preference for what the phase of synchrony will be. Our numerical results suggest that, in the case of anisotropic connections, for *U*(*t*) = {r ∈ ℝ^2^ | x ≤ ct} we have that *v*(*r*, *θ*, *t*) = *v*_*e*_(*x*, *θ*, *t*) where *v*_*e*_ is the 1D traveling bump solution, except that the center of the bump is at *θ* = *π*/2. It then follows from shift-twist invariance [60, 75] that for the rotated region $\bar{U}(t)=R_{\phi }U(t)$ the corresponding solution is given by *v*(*r*, *θ*, *t*) = *v*_*e*_(*R*_*ϕ*_ *x*, *θ* − *ϕ*, *t*), which has bump center at *θ* = *π*/2 + *ϕ*. Hence for waves in the deep layer traveling in the direction **k** ^⊥^ = (sin*ϕ*, cos*ϕ*) the corresponding activity in the superficial layer is a traveling bump with orientation coinciding with the direction **k**.

**Fig 10:**
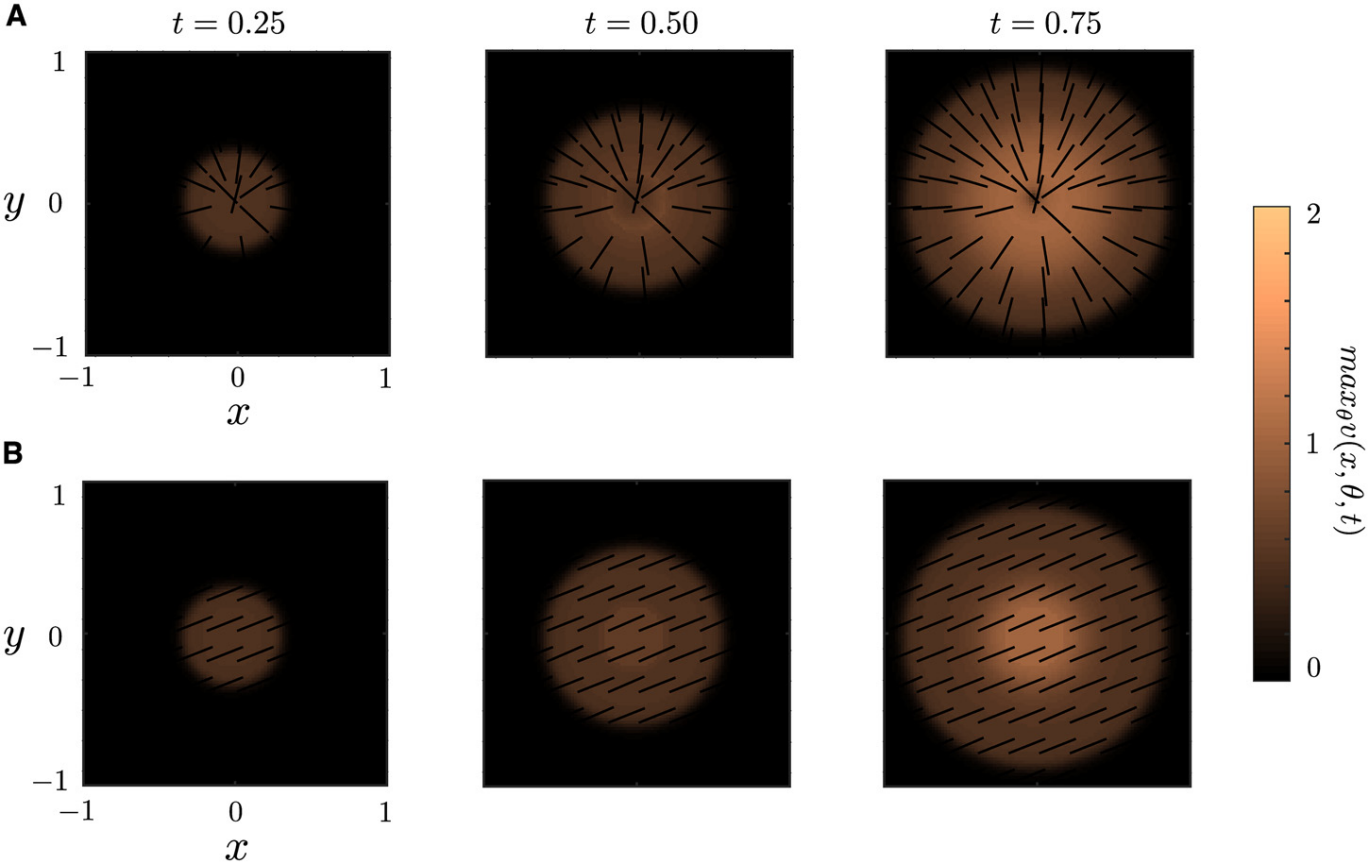
Two-dimensional target patterns. Numerical simulation of Eqs (4a) and (4b) for **(A)** anisotropic connection *A*(*θ*) = 1 + cos(2*θ*) and **(B)** isotropic connections *A* ≡ 1. All parameter values are the same as in Fig 6 and we take *r*_0_ = 0.1. All weight functions are the 2D analogs of the functions used for 1D.

Our second and final example demonstrates the dynamics when the deep layer supports a traveling wave solution with a circular front. Such a solution is given by a target wave [76–79]. In this case the region is given *U*(*t*) = {r ∈ ℝ^2^|0 ≤ |r| ≤ *ct*}. In the stationary case, *U*(*t*) = {r ∈ ℝ^2^|0 ≤ |r| ≤ *R*} for any *R*, we found that the angular solution *ϕ*_*s*_(*r*) = arg(*r*) is a solution for both isotropic and anisotropic connections, whereas the synchronous solution only exists for isotropic connections. We find that the results are analogous in the traveling wave scenario. In Fig 10, we show a numerical simulation with initial conditions

$$u\left(r,0\right)=H(r_{0}-|r|),\;v\left(r,\theta ,0\right)=0.$$

We see that with anisotropic connections the phase of the bumps tend towards the angular solution *ϕ*(*r*) = arg(*r*), suggesting that the anisotropic connections along with the geometry of the region determine the phase structure of the solution. On the other hand, with isotropic connections we see that there is no such structure in the phase. Rather, it appears that the solution tends towards a synchronous solution.

## Discussion

In this paper we have considered two important features of visual cortex that are usually neglected when developing neural field models of cortical waves, namely, the laminar structure of cortex and the orientation preferences of V1 cells in superficial layers. The latter naturally leads to the introduction of neural fields on product spaces [60, 61, 80]. It turns out that obtaining an orientation-dependent traveling front solution of the laminar model is non-trivial, requiring a delicate geometric construction. Two major predictions of our model are (i) long-range horizontal connections in superficial layers play a crucial role in supporting orientation-dependent propagating waves, and (ii) the waves originate in deep layers and are not strongly affected by feedback from superficial layers.

It is important to emphasize that we made a number of assumptions regarding the functional architecture of V1, which are still not settled experimentally and are likely to depend on the particular animal model. These assumptions include the following:

1. Neurons in deep layers are weakly tuned for orientation. In particular, orientation tuning only occurs in response to oriented visual stimuli, but does not arise spontaneously due to a lack of orientation-dependent recurrent connections.
2. Feedback from superficial to deep layers is orientation-independent.
3. Traveling waves can occur in an isolated deep layer but not in an isolated superficial layer.
4. There is a direct feedforward drive from the deep layer to the superficial layer, which is necessary in order to observe propagating activity in the superficial layer.
5. Sharpening of orientation tuning occurs in superficial layers due to local recurrent excitation and lateral inhibition. Long-range connections are assumed to be excitatory.

It should be noted that advances in the use of viruses [81] means that it should eventually be possible to more accurately test these various assumptions by inactivating specific neuron populations in specific layers, and observing the effects on propgating activity. One possible application of the theory developed in this paper is to the study of binocular rivalry waves in V1 [13, 14, 82]. Binocular rivalry is the phenomenon where perception switches back and forth between different images presented to the two eyes; this switch does not occur simultaneously across the visual field but spreads like a propagating front. Previously, we developed a model of binocular rivalry waves consisting of two mutually-coupled 1D neural fields that were driven by left-eye and right-eye stimuli, respectively. Recurrent connections within each 1D network were assumed to be excitatory, whereas connections between the two networks were inhibitory (cross-inhibition). We showed that incorporating some form of slow adaptation such as synaptic depression into the model broke the symmetry between the left and right eye neural fields, thus allowing a front to propagate [15, 16]. Such a front represents the neural correlate of the propagating perceptual switch from one eye’s image to the other. One simplification of our previous work was to ignore the orientation preference of the neurons. However, it has been observed experimentally that the speed of binocular rivalry waves depends on the orientation of the left and right eye stimuli [13]. This suggests replacing the single layer left-eye and right-eye neural fields by a pair of laminar neural fields similar to the one considered in this paper.

Another possible extension of our work would be to consider other types of spatiotemporal patterns arising in a laminar neural field. For example, suppose that the deep-layer exhibited a Turing-like instability due to an increase in excitability, resulting in the spontaneous formation of a spatially periodic pattern. What would be the corresponding pattern of orientation-dependent in the superficial layer? This could provide a laminar-based extension of the theory of geometric visual hallucinations [60, 83].

Finally, it would be interesting to explore other aspects of how the functional architecture of cortex influences wave propagation. For example, by incorporating other feature preference maps such as ocular dominance (left/right eye preference) and spatial frequency [84] or variables associated with texture processing [85].) In doing so it might be necessary to distinguish between pinwheels and linear zones of the orientation preference map. It is important to note that the coupled ring model does not explicitly incorporate pinwheels. Hence, it cannot determine possible fine-scale differences between activity propagating near pinwheels and linear zones. On the other hand, the ring model itself can be interpreted as describing changes in orientation preference in linear zones as one rotates around a pinwheel.

## Materials and Methods

We present the details of our analysis of an orientation-dependent traveling wave solution of the neural field Eq (12), based on the graphical construction shown in Fig 8. We then show how feedback does not modify the speed of a traveling wave in the deep layer. Finally, we briefly summarize our numerical methods.

### Graphical construction of traveling wave in the superficial layer

We seek a solution of Eq (12) of the form *v*(*x*, *θ*, *t*) = *V*(*z*, *θ*) with *z* = *x* − *ct* and

$$\begin{array}{c} \lim_{z\to -\infty }V\left(z,\theta \right)=V_{\text{bump}}\left(\theta \right),\;\lim_{z\to \infty }V\left(z,\theta \right)=0, \end{array}$$ (14)

where *V*_bump_ satisfies

$$V_{\text{bump}}\left(\theta \right)=\left(w_{\text{loc}}\left(\theta \right)+w_{s,0}w_{\text{hoz}}\left(\theta \right)\right)*H_{s}\left(V_{0}\left(\theta \right)\right)+\gamma_{d}$$

for

$$w_{s,0}=\int_{-\infty }^{\infty }w_{s}\left(z\right)dz,$$

with *w*_loc_(θ) + *w*_s,0_ *w*_hoz_(θ) satisfying conditions (i)-(iii) below Eq (11). Substituting the wave solution into Eq (12) gives

$$\begin{array}{c} -c\partial_{z}V\left(z,\theta \right)=-V\left(z,\theta \right)+w_{\text{loc}}\left(\theta \right)*H_{s}\left(V\left(z,\theta \right)\right)+w_{s}\left(z\right)\circ w_{\text{hoz}}\left(\theta \right)*H_{s}\left(V\left(z,\theta \right)\right)+\gamma H\left(-z\right), \end{array}$$ (15)

where we have absorbed the *τ*_*s*_ term into *c* for notational clarity. If such a solution exists then there must be a point *z*_0_ < 0 such that *V*(*z*, *θ*) < *κ*_*s*_ for all (*z*, *θ*) ∈ (*z*_0_, ∞) × [−*π*/2, *π*/2]. That is there must be a point where the solution first reaches threshold. Furthermore this point must be negative since *γH*(−*z*) = 0 for *z* > 0 and hence the dynamics remains below threshold in this region, as we show more explicitly below. We then observe that for *z* > *z*_0_

$$\begin{array}{c} -c\partial_{z}V\left(z,\theta \right)=-V\left(z,\theta \right)+w_{s}\left(z\right)⊙w_{\text{hoz}}\left(\theta \right)*H_{s}\left(V\left(z,\theta \right)\right))+\gamma H\left(-z\right), \end{array}$$ (16)

and for *z* < *z*_0_

$$\begin{array}{c} c\partial_{z}V\left(z,\theta \right)=-V\left(z,\theta \right)+w_{\text{loc}}\left(\theta \right)*H_{s}\left(V\left(z,\theta \right)\right)+w_{s}\left(z\right)⊙w_{\text{hoz}}\left(\theta \right)*H_{s}\left(V\left(z,\theta \right)\right)+\gamma \end{array}$$ (17)

where

$$\begin{array}{c} w_{s}\left(z\right)⊙w_{hoz}\left(\theta \right)*H_{s}\left(V\left(z,\theta \right)\right)=\int_{-\infty }^{z_{0}}\int_{-\pi /2}^{\pi /2}w_{s}\left(z-z\prime \right)w_{hoz}\left(\theta -\theta \prime \right)H_{s}\left(V\left(z\prime ,\theta \prime \right)\right)d\theta \prime dz\prime . \end{array}$$ (18)

Therefore, the region *z* > *z*_0_ is only dependent on the activity in region *z* < *z*_0_ while the active region receives no input from the inactive region and is thus not dependent on the solution in the inactive region. In the following we let *V* ^+^(*z*, *θ*) denote the solution for *z* < *z*_0_ and *V* ^−^(*z*, *θ*) denote the solution for *z* > *z*_0_.

Using the existence of the point *z*_0_, we now determine the quiescent, queuing, integrating, and bump regions shown in Fig 8. In order to facilitate the analysis, we assume that *w*_*s*_ has compact support, say

$$w_{s}\left(z\right)=0,\;\left|z\right|>L,$$

such that ∣*z*_0_∣ < *L*. Then for *z* > *L*−∣*z*_0_∣ we have that

$$w_{s}\left(z\right)⊙w_{\text{hoz}}\left(\theta \right)*H_{s}\left(V\left(z,\theta \right)\right)\equiv 0,$$

and thus

$$\begin{array}{c} -c\partial_{z}V^{-}\left(z,\theta \right)=-V^{-}\left(z,\theta \right). \end{array}$$ (19)

Hence, the requirement that *V*(*z*, *θ*) → 0 as *z* → ∞ translates to *V* ^−^(*z*, *θ*) ≡ 0 for all *z* > *L* − ∣*z*_0_∣ and we call this the *quiescent region*.

For 0 < *z* < *L* − ∣*z*_0_∣

$$-c\partial_{z}V^{-}\left(z,\theta \right)=-V^{-}\left(z,\theta \right)+w_{s}\left(z\right)⊙w_{\text{hoz}}\left(\theta \right)*H_{s}\left(V^{+}\left(z,\theta \right)\right).$$

In this *queuing region*, the populations have not received input from the deep layer but receives a weak synaptic input from the active region. If *V* ^+^(*z*, *θ*) have the same bump center for all *z*, then *V* ^−^(*z*, *θ*) will also have the same center and will remain below threshold. More precisely, suppose that there is a unique *θ*_0_ such that

$$V^{+}\left(z,\theta_{0}\right)=\max_{\theta \in [-\frac{\pi }{2},\frac{\pi }{2}]}V^{+}\left(z,\theta \right)$$

for all *z* < 0. Then since *V* ^+^(*z*, *θ*) must be symmetric about *θ*_0_ we have that *V* ^+ ^(*z*, *θ*) > *κ*_*s*_ for *θ* ∈ (*θ*_0_ − Δ(*z*), *θ*_0_+Δ(*z*)) for each *z* > 0 and some function, Δ(*z*) describing the *z*-dependent bump half width. Hence multiplying both sides by $\frac{1}{c}e^{-z/c}$ and integrating we obtain, using the initial condition *V* ^−^(*L*−∣*z*_0_∣, *θ*) = 0,

$$V^{-}\left(z,\theta \right)=\frac{1}{c}\int_{L-\left|z_{0}\right|}^{z}e^{-\left(z^{\prime }-z\right)/c}\int_{-\infty }^{z_{0}}w_{s}\left(z\prime -z\prime \prime \right)\Omega_{hoz}\left(\theta -\theta_{0},\Delta \left(z\prime \prime \right)\right)dz\prime \prime dz\prime ,$$

where

$$\begin{array}{c} \Omega_{\text{hoz}}\left(\theta ,\Delta \right)=W_{\text{hoz}}\left(\theta +\Delta \right)-W_{\text{hoz}}\left(\theta -\Delta \right), \end{array}$$ (20)

with

$$W_{hoz}\left(\theta \right)=\int_{0}^{\theta }w_{hoz}\left(\theta \prime \right)d\theta \prime .$$

Thus the location of the max of *V* ^−^(*z*, *θ*), with respect to *θ*, is determined by the location of the max of Ω_hoz_(*θ* − *θ*_0_, Δ(*z*)), which is *θ*_0_. Furthermore, in this region the activity remains below threshold for sufficiently small *w*_s_. To see this it is simple to show that

$$|V^{-}\left(z,\theta \right)|\leq w_{s,0}w_{\text{hoz},0}(1-e^{-(L-|z_{0}|-z)/c})<w_{s,0}w_{\text{hoz},0}$$

for all 0 < *z* < *L*−∣*z*_0_∣. Hence, as long as *w*_s,0_ *w*_hoz,0_ < *k*_s_ then the solution remains below threshold. Thus, before the populations receive input, they are queued up as sub-threshold bumps to start integrating to super-threshold bumps with the appropriate center. This is the most important region with respect to propagation and is where horizontal connections play a crucial role. Without horizontal connections there is no queuing, and the solution remains at *V*(*z*, *θ*) = 0 until *z* = 0. Once the populations receive input, they start integrating towards the threshold state (unless perturbed by orientation-dependent noise), as described for the case *w*_*s*_ ≡ 0. However, with horizontal connections the super-threshold activity forms an orientation-dependent sub-threshold bump which provides a mechanism for both the destabilization of the threshold state as well as correlating the bumps in a way that the bump center remains the same as it propagates.

In the *integrating region* *z*_0_ < *z* < 0, we have

$$-c\partial_{z}V^{-}\left(z,\theta \right)=-V^{-}\left(z,\theta \right)+w_{s}\left(z\right)⊙w_{\text{hoz}}\left(\theta \right)*H_{s}\left(V^{+}\left(z,\theta \right)\right)+\gamma .$$

The populations now receive orientation-independent input from the deep layer and a weak orientation-dependent input from horizontal connections. Since the zero state no longer exists the resulting solution evolves as a subthreshold bump state until it reaches threshold at *z* = *z*_0_. Finally, in the *bump region* *z* < *z*_0_, the remaining dynamics are governed by the equation

$$\begin{array}{c} -c\partial_{z}V^{+}\left(z,\theta \right)=-V^{+}\left(z,\theta \right)+w_{\text{loc}}\left(\theta \right)*H_{s}\left(V^{+}\left(z,\theta \right)\right)+w_{s}\left(z\right)⊙w_{\text{hoz}}\left(\theta \right)*H_{s}\left(V^{+}\left(z,\theta \right)\right)+\gamma . \end{array}$$ (21)

Here, the solution integrates beyond threshold and continues to evolve towards the stationary bump.

### Feedback has no affect on wavespeed in the deep layer

We show that, in the case of Heaviside firing rate functions, the feedback connections do not affect the wave speed, rather only the shape of the wave. Traveling wave solutions in the deep layer with feedback satisfy

$$-c\partial_{z}U\left(z,\theta \right)+U\left(z,\theta \right)=w_{d}\left(z\right)\circ H_{d}\left(U\left(z,\theta \right)\right)+\gamma_{s}\int_{-\pi /2}^{\pi /2}H_{s}\left(V\left(z,\theta \right)\prime \right)d\theta \prime .$$

Following the analysis of bump solutions in **Results**, see also Fig 5, we look for traveling wave solutions *U*(*z*, *θ*) and *V*(*z*, *θ*) such that

$$\lim_{z\to \infty }U\left(z,\theta \right)=\lim_{z\to \infty }V\left(z,\theta \right)=0$$

and

$$\lim_{z\to -\infty }U\left(z,\theta \right)=w_{d,0}+2\Delta \gamma_{s},\;\lim_{z\to -\infty }V\left(z,\theta \right)=V_{\text{bump}}\left(\theta \right)=W\left(\theta +\Delta \right)-W\left(\theta -\Delta \right)+\gamma_{d}$$

**Fig 11:**
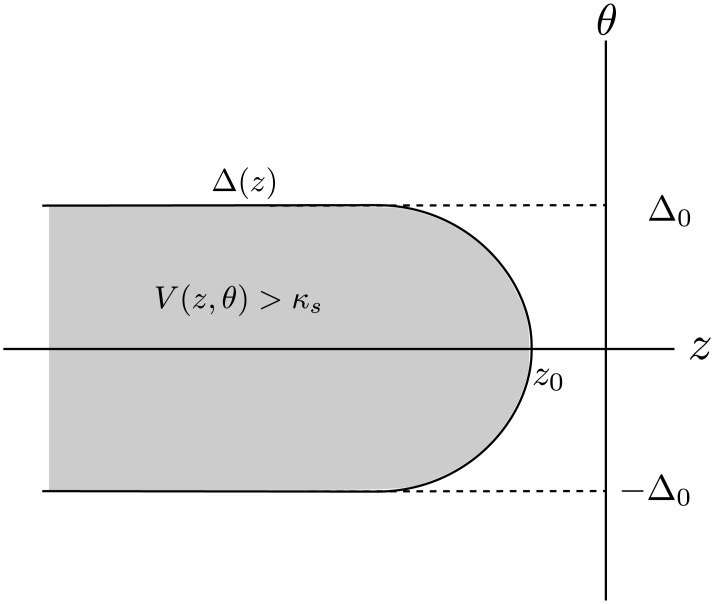
Schematic illustration of superficial layer traveling front solution *V*(*z*, *θ*) in the z-*θ* plane. The curve determined by ±Δ(*z*) separates the plane into a region of super threshold activity (grey) and sub threshold activity.

Note that the asymptotic condition for the up state in the deep layer now includes a feedback term 2Δ*γ*_*s*_ where Δ is the half-width of the bump solution *V*_bump_. Assuming that *V*(*z*, *θ*) is a traveling wave, there must exist some point *z*_0_ such that the solution first reaches threshold. Moreover, if *w*_s,0_ is sufficiently small, so as to not support propagation on its own, then *z*_0_ < 0 necessarily (see Fig 11). That is, the threshold crossing for the superficial layer lags behind the deep layer, and beyond this point the solution integrates towards a bump solution. Thus at each *z* < *z*_0_ there is a function describing the instantaneous bump width, Δ(*z*) with the conditions

$$\Delta \left(z_{0}\right)=0,\;\lim_{z\to -\infty }\Delta \left(z\right)=\Delta_{0}.$$

This function describes the threshold crossing contour, i.e. *V*(±Δ(*z*), *z*) = *κ*_*s*_. We can then write *H*_*s*_(*V*(*z*, *θ*)) = *H*(Δ(*z*)−∣*θ*∣)*H*(*z*_0_−*z*). Plugging this form into the integral yields

$$\int_{-\pi /2}^{\pi /2}H_{s}\left(V\left(z,\theta \prime \right)\right)d\theta \prime =H\left(z_{0}-z\right)\int_{-\pi /2}^{\pi /2}H(\Delta \left(z\right)-\left|\theta \right|)d\theta =2H\left(z_{0}-z\right)\Delta \left(z\right).$$

Therefore, when carrying out the computation for the positive wave speed as above we obtain

$$\begin{array}{ccc} \kappa_{d} & = & \frac{1}{c}\int_{0}^{\infty }e^{-y/c}[W_{d}\left(y\right)+2\gamma_{s}H\left(z_{0}-y\right)\Delta \left(y\right)]dy\\ & = & \frac{1}{c}\int_{0}^{\infty }e^{-y/c}W_{d}\left(y\right)dy \end{array}$$

where the integration with the Heaviside term vanishes since it is identically zero for *z*_0_ < 0. Therefore the wave speed remains unchanged when adding feedback connections. This can be also understood by viewing neural fields in terms of interface dynamics as in [86], where they show that with a Heaviside firing rate function, the evolution of solutions only depend on the activity at the interface (or the threshold crossings). Calculating the solution yields

$$\begin{array}{ccc} U(z,\theta ) & = & \frac{1}{c}\int_{z}^{\infty }e^{-y/c}\left[W_{d}\left(y\right)+2\gamma_{s}H\left(z_{0}-y\right)\Delta \left(y\right)\right]dy\\ & = & \frac{1}{c}\int_{z}^{\infty }e^{-y/c}W_{d}\left(y\right)dy+2\gamma_{s}H\left(z_{0}-z\right)\int_{z}^{z_{0}}e^{-y/c}\Delta \left(y\right)dy\\ & = & U_{0}\left(z\right)+2\gamma_{s}H\left(z_{0}-z\right)\int_{z}^{z_{0}}e^{-y/c}\Delta \left(y\right)dy \end{array}$$

where *U*_0_(*z*) is the traveling wave solution in the absence of feedback. Since Δ(*z*) ≥ 0 and *γ*_*s*_ > 0 the second integral term is positive for *z* < *z*_0_ and identically zero for *z* ≥ *z*_0_. Therefore the threshold crossing for *U*_0_(*z*) and *U*(*z*, *θ*) coincide and thus *H*_*d*_(*U*(*z*)) = *H*_*d*_(*U*_0_(*z*)) = *H*(−*z*). Hence, the dynamics in the superficial layer become

$$-cV\left(z,\theta \right)+V\left(z,\theta \right)=w_{\text{loc}}\left(\theta \right)*H_{s}\left(V\left(z,\theta \right)\right)+w_{s}\left(z\right)\circ w_{\text{hoz}}\left(\theta \right)*H_{s}\left(V\left(z,\theta \right)\right)+\gamma_{d}H\left(-z\right),$$

which is precisely what one obtains when ignoring feedback, i.e. setting *γ*_*s*_ = 0. A mathematically convenient consequence of this fact is that we can first prove existence of traveling wave solutions in the deep layer, in the absence of feedback, independently of the dynamics in the superficial layer. We can then use this solution to provide an effective time-dependent input to the superficial layer, and analyze its dynamics independently of the deep layer. Note, however, that this is purely an artifact of the Heaviside firing rate and in general may not apply to arbitrary firing rate functions. However, in the *Results*, we show numerically that the wave properties persist when using a smooth firing rate with sufficiently large gain, *η*.

### Numerical methods

All numerical simulations were performed in Matlab. One dimensional numerical simulations were performed using a forward Euler method scheme in time and a trapezoidal rule for integration in *x* and *θ*. Time steps were taken to be Δ*t* = 0.001, spatial steps Δ*x* = 0.01 and orientation steps Δ*θ* = 0.01*π*. The numerical methods employed for 2D simulations were similar. However, rather than using the trapezoidal rule for integration in *x*, we used Fast Fourier Transforms to compute the convolutions. Recall that the convolution theorem states

$$f*g=F^{-1}(F[f]F[g]),$$

where f*g is the convolution of *f* and *g* and F[f] is the Fourier transform of *f*. We can therefore write the neural field equation

$$\partial_{t}u=-u+w\left(x\right)*f\left(u\left(x,t\right)\right)$$

as

$$\partial_{t}u=-u+F^{-1}(F[w]F[f(u)]).$$

We therefore compute integration in *θ* using a trapezoidal rule for each fixed *x*. We then take the result and perform fast Fourier transforms to compute the remaining convolution in *x*. Time steps were taken as Δ*t* = 0.001, spatial steps Δ*x* = 0.02 and orientation steps Δ*θ* = 0.01*π*. To plot the vector fields we find a *ϕ*(*x*, *t*) such that

$$v\left(x,\phi ,t\right)=\max_{\theta }\;v\left(x,\theta ,t\right)$$

at each *x*, *t*, which denotes the tuned orientation of the population. We then define the vector field as

v(x,t)=(cos(ϕ(x,t),sin(ϕ(x,t)).
